# *XRCC3* C18067T Polymorphism Contributes a Decreased Risk to Both Basal Cell Carcinoma and Squamous Cell Carcinoma: Evidence from a Meta-Analysis

**DOI:** 10.1371/journal.pone.0084195

**Published:** 2014-01-15

**Authors:** Xu Chen, Zhe Wang, Yulan Yan, Ping Li, Zheng Yang, Lingyan Qin, Wuning Mo

**Affiliations:** 1 Department of Clinical Laboratory, First Affiliated Hospital of Guangxi Medical University, Nanning, Guangxi, People's Republic of China; 2 Division of Spine and Osteopathy surgery, The First Affiliated Hospital of Guangxi Medical University, Nanning, Guangxi, People's Republic of China; University of Connecticut Health Center, United States of America

## Abstract

**Background:**

The X-ray repair cross-complementing group 3 (XRCC3) in homologous recombination repair (HRR) pathway plays a very important role in DNA double-strand break repair (DSBR). Variations in the *XRCC3* gene might lead to altered protein structure or function which may change DSBR efficiency and result in cancer. The *XRCC3* C18067T polymorphism has been reported to be associated with skin cancer susceptibility, yet the results of these previous results have been inconsistent or controversial. To derive a more precise estimation of the association, we conducted a meta-analysis.

**Methods:**

The quality of the studies was assessed according to a predefined scale. The association between the *XRCC3* C18067T polymorphism and skin cancer risk was assessed by odds ratios (ORs) together with their 95% confidence intervals (CIs).

**Results:**

Overall, no significant association was observed between *XRCC3* C18067T polymorphism and skin cancer risk in any genetic model. Stratified analyses according to tumor type, significant association was found in the relationship between *XRCC3* C18067T polymorphism and nonmelanoma skin cancer risk (homozygote comparison TT versus CC: OR = 0.74, 95%CI = 0.61–0.90, *P* = 0.003; recessive model TT versus TC/CC: OR = 0.81, 95%CI = 0.68–0.95, *P* = 0.01). Furthermore, significant association was also observed in *XRCC3* C18067T polymorphism with both basal cell carcinoma risk (homozygote comparison TT versus CC: OR = 0.70, 95%CI = 0.53–0.92, *P* = 0.011; recessive model TT versus. TC/CC: OR = 0.74, 95%CI = 0.60–0.92, *P* = 0.007) and squamous cell carcinoma risk (heterozygote comparison TT versus .CC: OR = 0.81, 95%CI = 0.67–0.99, *P* = 0.04; dominant model TT/TC versus .CC: OR = 0.81, 95%CI = 0.68–0.98, *P* = 0.029).

**Conclusion:**

The present meta-analysis demonstrates that *XRCC3* C18067T polymorphism was not associated with risk of cutaneous melanoma but contributed a decreased risk to both basal cell carcinoma and squamous cell carcinoma.

## Introduction

Skin cancer is one of the most frequent malignant diseases in humans, especially in the Western world [1]. More than two million cases of skin cancer are diagnosed in the USA every year [2]. There are several main subtypes of skin cancer including cutaneous melanoma and nonmelanoma skin cancer (NMSC) which consists of basal cell carcinoma (BCC) and squamous cell carcinoma (SCC). During the past decades, its incidence has increased globally, which makes a negative effect on human health [3]. Therefore, it is of great importance to get a thorough knowledge of skin cancer, especially its etiology.

Extensive epidemiological, experimental, and molecular evidence indicates UV radiation as an important environmental carcinogen involved in the initiation and progression of skin cancer [4]. Many studies have demonstrated that the incidence of skin cancer varies greatly between different countries and different ethnicities, suggesting genetic factors play important roles in the development of skin cancer [5]–[7]. It is well accepted that UV radiation could cause various kinds of DNA damage and the cellular response to DNA damage promotes activation of many DNA repair pathways involving dozens of genes with unique repair functions. Therefore, the DNA repair systems play an important role in maintaining the integrity of the genome and protecting against mutations that can lead to cancer, including skin cancer.

The X-ray repair cross-complementing group 3 (XRCC3) protein, involved in the homologous recombination repair (HRR) pathway, is a member of an emerging family of Rad-51-related proteins participating in HRR to repair DNA damage and maintain genomic stability [8]. XRCC3-deficient cells presented defects in Rad51 focus formation after radiation damage and demonstrated genetic instability and increased sensitivity to DNA damaging agents [9]. So that *XRCC3* has been of great interest as a candidate gene for cancers, including skin cancer.

The *XRCC3* gene is located in human chromosomes 14q32.3 and various polymorphisms in this gene have been identified with susceptibility to cancers such as Thr241Met (C18067T, rs861539), 5-UTR (A4541G rs1799794) and IVERSUS 5–14 (A17893G, rs1799796). In the past decade, the majority of molecular epidemiologic studies investigated *XRCC3* C18067T polymorphism on skin cancer susceptibility. However, the results remain fairly conflicting rather than conclusive. To derive a more precise estimation of the relationship between *XRCC3* C18067T polymorphism and skin cancer risk, we conducted a meta-analysis of all available case-control studies relating the *XRCC3* C18067T polymorphism to the risk of developing skin cancer.

## Materials and Methods

### Search strategy

This study was conducted based on the predefined proposal of Meta-analysis of Observational Studies in Epidemiology group [10]. We performed a comprehensive literature search in PubMed, EMBASE and Chinese Biomedical Literature Database (CBM) using the terms as follows: “X-ray repair cross-complementing group 3 or *XRCC3* or DSBR” in combination with “polymorphism or variant or mutation” and in combination with “Skin cancer” updated on July 2013 for all publications on the association between *XRCC3* C18067T polymorphism and skin cancer risk. There was no restriction on time period, sample size, population, language, or type of report. All eligible studies were retrieved, and their bibliographies were checked for other relevant publications. Additional studies were identified by a hand search of the references of original studies. The literature retrieval was performed in duplication by two independent reviewers (Xu Chen and Zhe Wang). When a study reported the results on different subpopulations, we treated it as separate studies in the meta-analysis.

### Inclusion and Exclusion criteria

The following inclusion criteria were used for the paper selection: (a) a case-control study; (b) information on the relationship between *XRCC3* C18067T polymorphisms and skin cancer risk; (c) the papers had to provide the size of the samples, distribution of alleles, genotypes or other information that can help us infer the results; (d) Of the studies with overlapping data published by the same investigators, we chose the most recent or complete study was included. Accordingly, studies were excluded if one of the following existed: (a) studies that contained overlapping data; (b) Not offering the source of cases and controls or other essential information; (c) studies in which family members had been studied because their analysis are based on linkage considerations.

### Data extraction

All the data were extracted by two investigators (Xu Chen and Zhe Wang) independently with the standard protocol and the result was reviewed by a third investigator (Yulan Yan). From each study, we extracted the name of first author, year of publication, country of origin, ethnicity of the population studied, the number of cases and controls, allele frequency, definition of cases, and genotype distribution in cases. Different ethnic descents were categorized as Caucasians, Asian, African or Mixed. For studies including subjects of different ethnic groups, data were extracted for ethnic group whenever possible. To ensure the accuracy of the extracted information, two investigators (Xu Chen and Zhe Wang) checked the data extraction results and reached consensus on all of the data extracted. If different results generated, they would check the data again and have a discussion to come to an agreement. A third reviewer (Yulan Yan) was invited to the discussion if disagreement still existed.

### Methodological quality assessment

Methodological quality of eligible studies was also independently assessed by the same two reviewers (Xu Chen and Zhe Wang) according to a set of predefined criteria (Table 1) mainly based on the scale of Jiang et al [11]. These scores were based on both traditional epidemiological considerations and cancer genetic issues. Disagreements were resolved by consensus. Scores ranged from 0 (lowest) to 18 (highest). Articles with scores less than 12 were considered “low-quality” studies, whereas those with scores equal to or higher than 12 were considered “high-quality” studies.

**Table 1:** Scale for Quality Assessment.

Criterion	Score
Representativeness of cases	
Selected from population or cancer registry	3
Selected from hospital	2
Selected from pathology archives, but without description	1
Not described	0
Credibility of controls	
Population-based	3
Blood donors or volunteers	2
Hospital-based (cancer-free patients)	1
Not described	0
Ascertainment of skin cancer	
Histologic or pathologic confirmation	3
Diagnosis of bladder cancer by patient medical record	1.5
Not described	0
Genotyping examination	
Genotyping done under “blinded” condition	3
Unblinded or not mentioned	0
Hardy-Weinberg equilibrium	
Hardy–Weinberg equilibrium in controls	3
Hardy–Weinberg disequilibrium in controls	0
Total sample size	
>1,000	3
>500 and ≤1,000	2
>200 and≤500	1
≤200	0

### Statistical analysis

The STATA Software (version 12.0, Stata Corp) was used to analyze the data. For each study, odds ratio (OR) and its 95% confidence interval (95%CI) were calculated to assess the association strength. The pooled ORs were performed in different genetic comparison models, including homozygote comparison (TT versus CC), heterozygote comparison (TC versus CC) recessive model (TT versus TC/CC), and dominant model (TT/TC versus CC).

The heterogeneity between the studies was assessed by the χ^2^-test based Q-statistic. A significant Q-statistic (P<0.10) suggested heterogeneity among studies, thus the summary OR estimate of each study was computed by the random-effects model [12]. Otherwise, the fixed-effects model was used [13]. In addition, I^2^ = 100%* (Q – df)/Q, was applied to assess heterogeneity between studies [14]. The I statistic measures the degree of inconsistency in the studies by calculating what percentage of the total variation across studies is due to heterogeneity rather than by chance [15].

The overall estimate of risk (OR) was calculated by a fixed effects model or a random effects model according to the presence (P<0.10 or I^2^>50%) or absence (P>0.10 and I^2^<50%) of heterogeneity, respectively. To better examine possible sources of between-study heterogeneity, meta-regression analysis was also used to both overall analyses and subgroup analyses when heterogeneity was observed. In addition, the Galbraith plot was applied to spot the outliers as the possible major sources of heterogeneity [16]. To validate the credibility of outcomes in this meta-analysis, the sensitivity analysis was also performed to identify potentially influential studies. The distribution of the genotypes in the control population was tested for Hardy–Weinberg equilibrium (HWE) using a goodness-of-fit Chi-square test.

Publication bias was observed and evaluated by the funnel plot [17] using the standard error of log (OR). An asymmetric plot suggests a possible publication bias. Funnel plot asymmetry was further evaluated by the method of Egger linear regression test [18]. The significance of the intercept was determined by the Student t test suggested by Egger (P<0.05 was considered representative of statistically significant publication bias).

## Results

### Eligible studies

**Figure 1:**
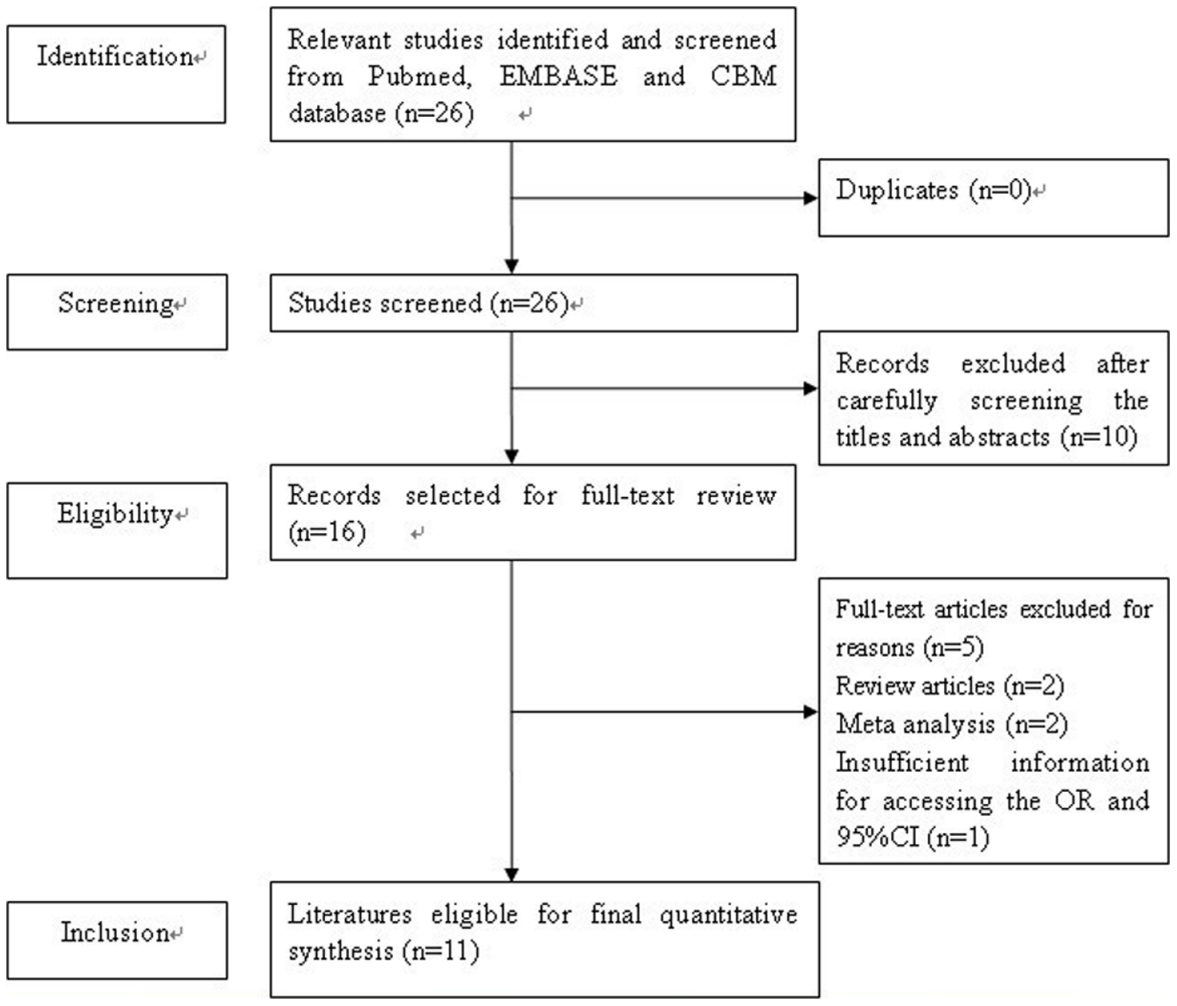
Flow diagram of included studies for this meta-analysis.

Based on the search criteria, 26 individual literatures were found. After screening the titles and abstracts, 10 studies were excluded because they were not relevant to the role of *XRCC3* C18067T polymorphism on skin cancer risk. Therefore, 16 full-text publications were preliminarily identified for further detailed evaluation (Figure 1). According to the exclusion criteria, 5 publications were excluded: 2 publications were review articles [19], [20], 1 for not presenting sufficient data of genotype or allelic for calculating OR and 95% CI [21], and 2 were meta-analysis [22], [23]. Manual search of references cited in the published studies did not reveal any additional articles. A flow diagram of the search process is shown in Figure 1.

As a result, a total of 11 relevant literatures which contain 15 case-control studies met the inclusion criteria for the meta-analysis [24]–[34]. The 15 case-control studies are comprised of more than 10000 individuals including 4329 cases and 7291 controls. The main characteristics of 15 case-control studies were presented in Table 2. Of 15 case-control studies, 14 were conducted in Caucasian populations and 1 was in Mixed population. Six studies were population-based and 9 were hospital-based studies. Seven studies investigate the skin cancer on the cutaneous melanoma, 5 studies on basal cell carcinoma and 3 studies on squamous cell carcinoma. Nine studies used age as the matching criteria between case groups and control populations and 4 studies in the current meta-analysis did not provide definite criteria for skin cancer conformation (histologically or pathologically confirmed). Three genetyping methods were applied in the present case-control studies such as PCR-RFLP, TaqMan Assay and PCR-SSP. All the genotype distributions of control population were consistent with HWE for *XRCC3* C18067T polymorphism.

**Table 2:** Characteristics of eligible studies.

First author (year)	Ethnicity (country)	Genotyping methods	Source of control	Sample size (case/control)	Skin cancer confirmation	Matching criteria	Tumor type	HWE(*P* value)	Quality score
Winsey (2000)	Caucasian (UK)	PCR-SSP	HB	125/211	HC	NR	melanoma	0.113	12
Duan (2002)	Caucasian (America)	PCR-RFLP	HB	305/319	HC	Age, sex ethnicity	melanoma	0.451	12
Jacobsen (2003)	Caucasian (Denmar)	PCR-RFLP	PB	318/318	NR	Age, sex	BCC	0.095	10
Bertram (2004a)	Caucasian (UK)	PCR-SSP	HB	140/362	NR	NR	melanoma	0.973	12.5
Bertram (2004b)	Caucasian (UK)	PCR-SSP	HB	140/203	NR	NR	melanoma	0.696	11.5
Han (2004a)	Caucasian (America)	TaqMan	PB	220/665	PC	Age	SCC	0.741	17
Han (2004b)	Caucasian (America)	TaqMan	PB	187/810	PC	Age and ethnicity	melanoma	0.359	17
Han (2004c)	Caucasian (America)	TaqMan	PB	276/810	PC	Age and ethnicity	SCC	0.359	18
Han (2004d)	Caucasian (America)	TaqMan	PB	279/810	PC	Age and ethnicity	BCC	0.359	18
Festa (2005)	Caucasian (Sweden)	PCR-RFLP	PB	197/548	NR	NR	BCC	0.541	10.5
Thirumaran (2006a)	Caucasian (Multiple)	TaqMan	HB	293/259	HC	Age, sex ethnicity	BCC	0.565	15
Thirumaran (2006b)	Caucasian (Multiple)	TaqMan	HB	237/274	HC	Age, sex ethnicity	BCC	0.565	15
Figl (2010)	Caucasian (German)	TaqMan	HB	1184/1274	HC	Ethnicity	melanoma	0.07	12
Gonçalves (2011)	Mixed (Brail)	PCR-RFLP	HB	192/192	HC	Age, sex	melanoma	0.788	11.5
Makowska (2012)	Caucasian (Poland)	PCR-RFLP	HB	236/236	HC	NR	SCC	0.968	10

HC, Histologically confirmed; PC, Pathologically confirmed; NR Not reported; PB, Population–based; HB, Hospital–based; HWE, Hardy–Weinberg equilibrium in control population; PCR–RFLP, Polymerase chain reaction-restriction fragment length polymorphism; PCR-SSP, Polymerase chain reaction-sequence-specific primer; BCC, basal cell carcinoma; SCC, squamous cell carcinoma.

### Quantitative synthesis of data

**Figure 2:**
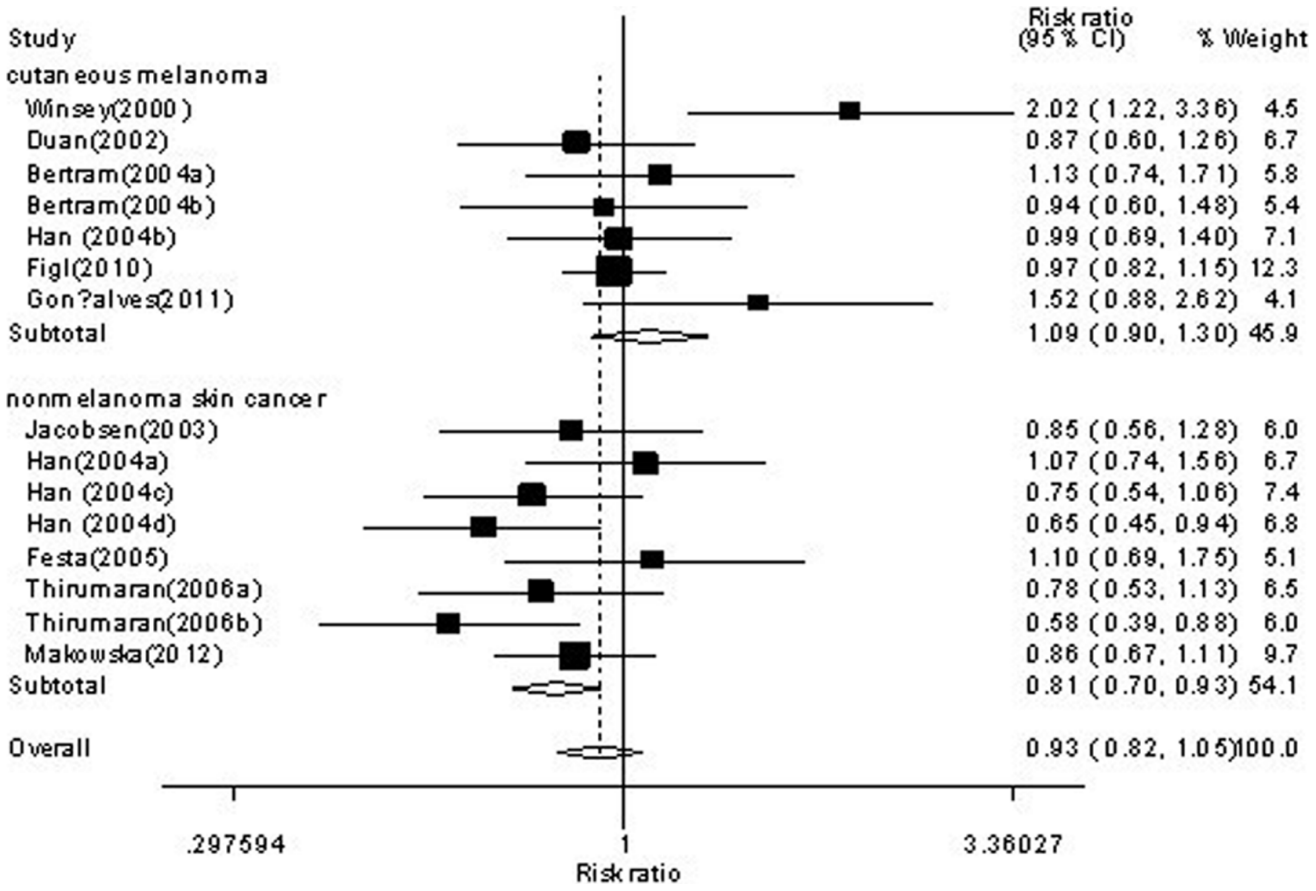
Forest plots of *XRCC3* C18067T polymorphisms and skin cancer risk which is stratified by cutaneous melanoma and nonmelanoma skin cancer (homozygote comparison TT versus . CC).

**Figure 3:**
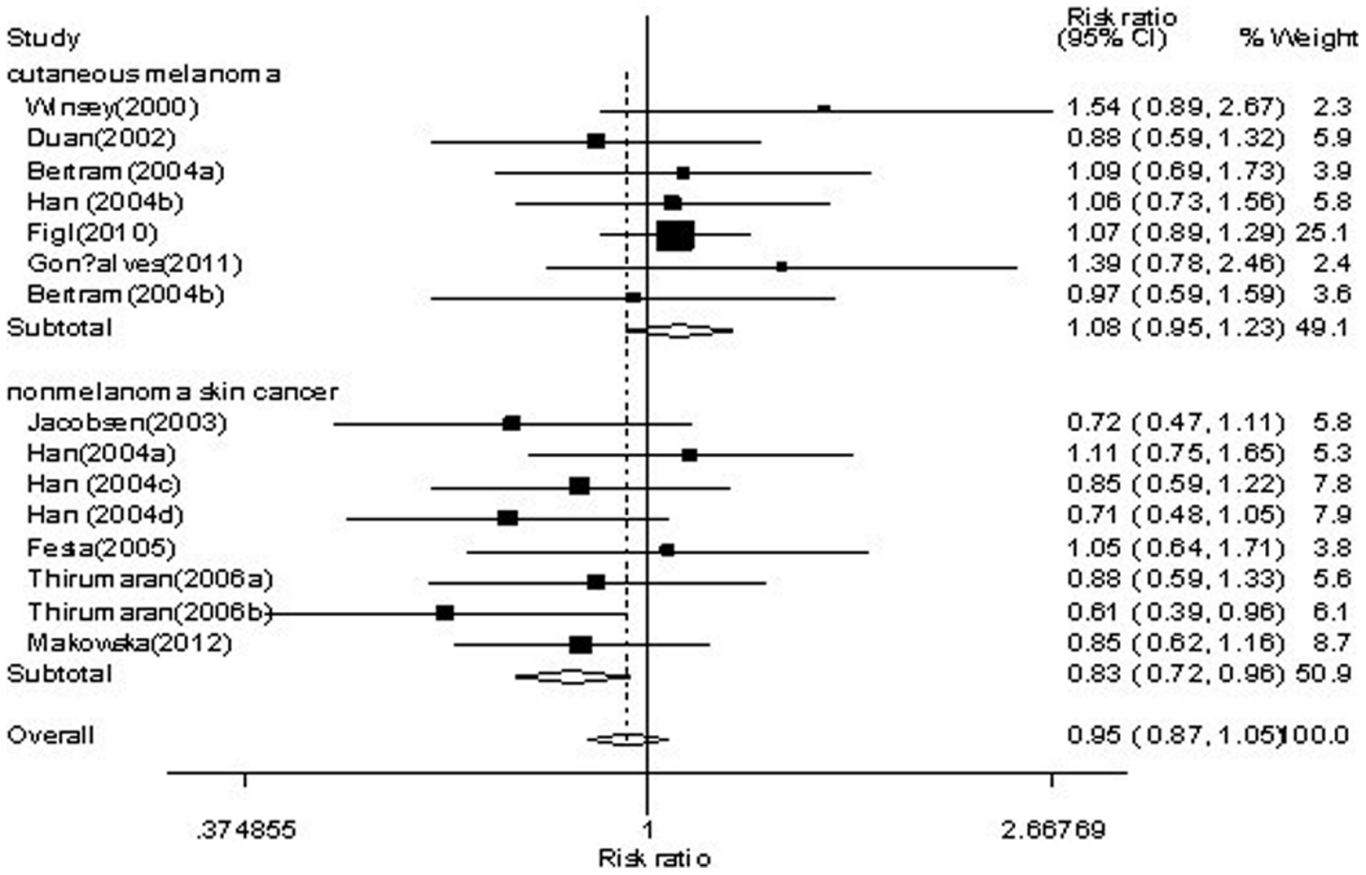
Forest plots of *XRCC3* C18067T polymorphisms and skin cancer risk which is stratified by cutaneous melanoma and nonmelanoma skin cancer (recessive model TT versus . TC+CC).

**Figure 4:**
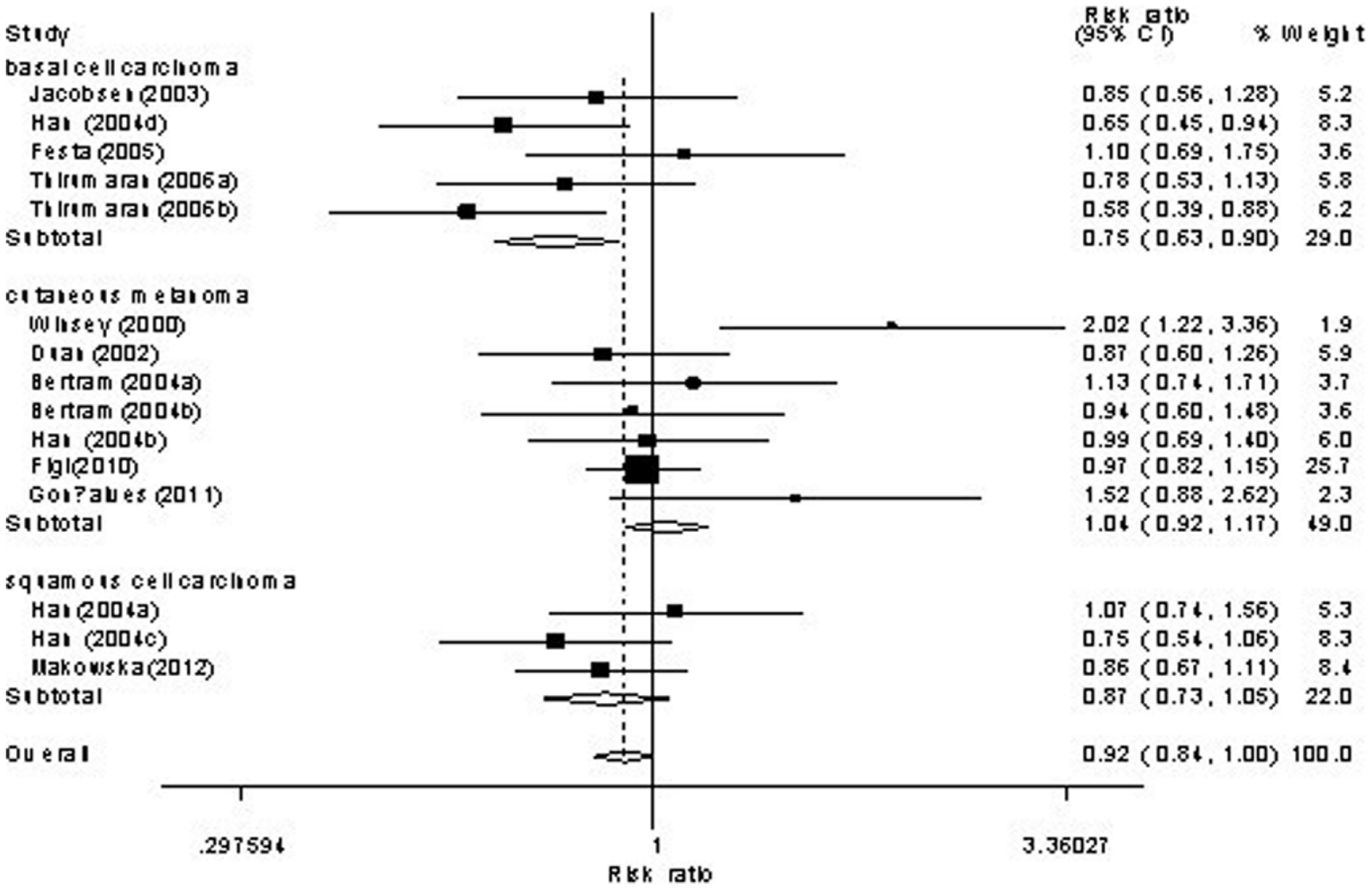
Forest plots of *XRCC3* C18067T polymorphisms and skin cancer risk which is stratified by cutaneous melanoma, basal cell carcinoma and squamous cell carcinoma (homozygote comparison TT versus . TC/CC).

**Figure 5:**
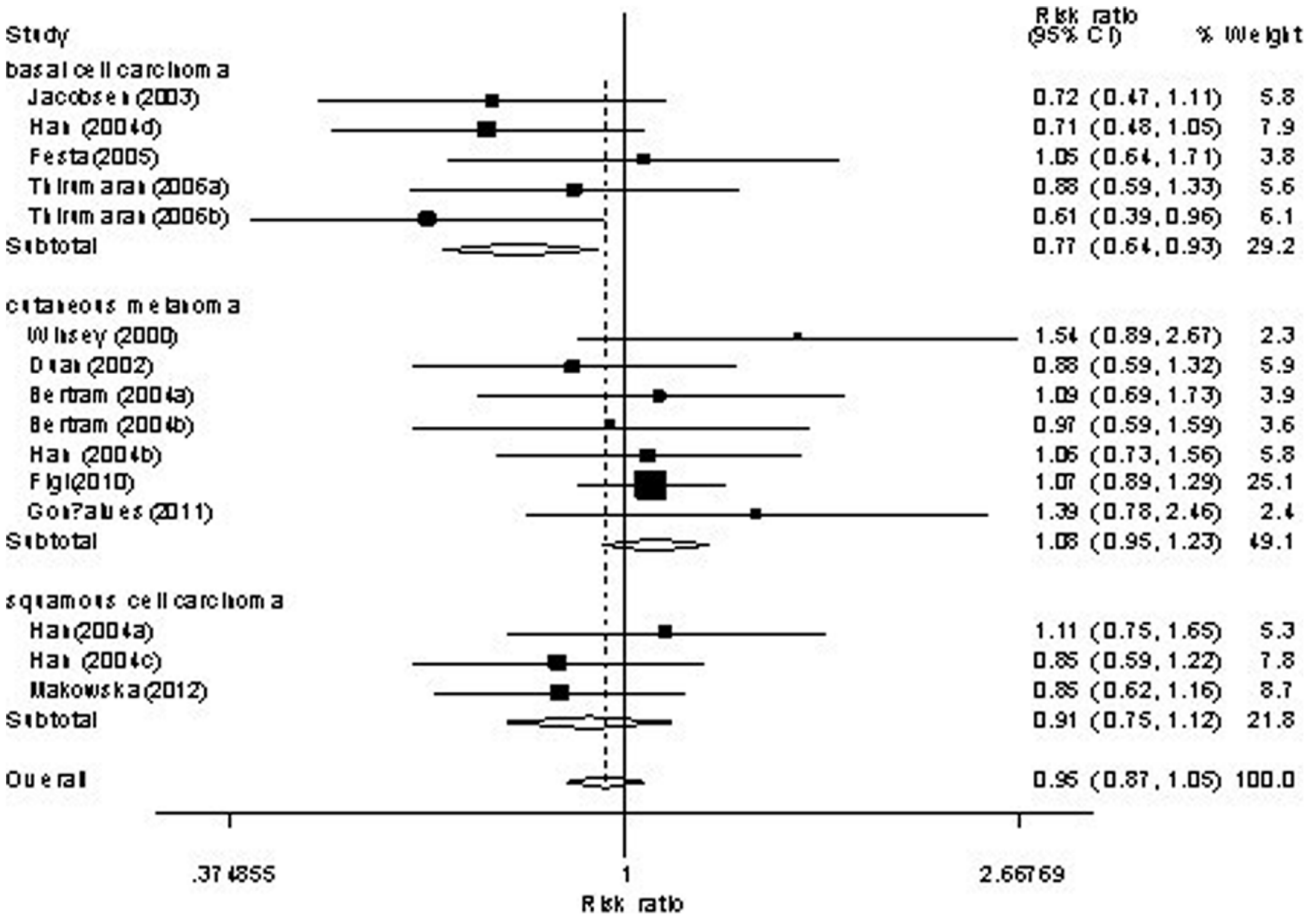
Forest plots of *XRCC3* C18067T polymorphisms and skin cancer risk which is stratified by cutaneous melanoma, basal cell carcinoma and squamous cell carcinoma (recessive model TT versus . TC/CC).

Forest plot of skin cancer risk associated with *XRCC3* C18067T polymorphism is shown under homozygote comparison (TT versus CC) in Figure 2 and receive model (TT versus TC/CC) in Figure 3. The meta-analysis showed insignificant association between skin cancer and *XRCC3* C18067T polymorphism in the overall population. In the stratified analysis by tumor type, the present meta-analysis showed that the C18067T polymorphism was associated with decreased nomelanoma risk (homozygote comparison TT versus. CC: OR = 0.74, 95%CI = 0.61–0.90, *P* = 0.003; Figure 2 and recessive model TT versus. TC/CC: OR = 0.81, 95%CI = 0.68–0.95, *P* = 0.01; Figure 3). Further subgroup analysis by subtype of nonmelanoma, we found that the *XRCC3* C18067T polymorphism contributed decreased risk to not only basal cell carcinoma (homozygote comparison TT versus. CC: OR = 0.70, 95%CI = 0.53–0.92, *P* = 0.011; Figure 4 and recessive model TT versus. TC/CC: OR = 0.74, 95%CI = 0.60–0.92, *P* = 0.007; Figure 5) but also squamous cell carcinoma (heterozygote comparison TT versus. CC: OR = 0.81, 95%CI = 0.67–0.99, *P* = 0.04; Figure not shown and dominant model TT/TC versus. CC: OR = 0.81, 95%CI = 0.68–0.98, *P* = 0.029; Figure not shown). The detailed results of the present meta-analysis were shown in Table 3.

**Table 3:** Meta-analysis of the XRCC3 C18067T polymorphism with risk of skin cancer.

Comparison	Tumor type	N	OR	95%CI	P_a_	Mode	χ2	P_b_	I^2^
TT versus. CC	Overall	15	0.90	0.75–1.07	0.242	Random	26.58	0.022	47.3
	melanoma	7	1.12	0.87–1.43	0.38	Fixed	10.12	0.120	40.7
	NMSC	8	**0.74**	**0.61–0.90**	**0.003**	Fixed	8.54	0.288	18
	BCC	5	**0.70**	**0.53–0.92**	**0.011**	Fixed	5.66	0.226	29.3
	SCC	3	0.81	0.61–1.08	0.158	Fixed	2.13	0.346	5.9
TC versus. CC	Overall	15	0.95	0.82–1.10	0.475	Random	37.72	0.001	62.9
	melanoma	7	1.07	0.84–1.37	0.593	Random	20.11	0.003	70.2
	NMSC	8	0.87	0.72–1.04	0.131	Random	16.33	0.022	57.1
	BCC	5	0.89	0.67–1.19	0.433	Random	13.98	0.007	71.4
	SCC	3	**0.81**	**0.67–0.99**	**0.040**	Fixed	1.83	0.401	0
TT versus. TC/CC	Overall	15	0.95	0.85–1.06	0.322	Fixed	16.32	0.294	14.2
	melanoma	7	1.09	0.94–1.28	0.256	Fixed	3.50	0.744	0
	NMSC	8	**0.81**	**0.68–0.95**	**0.01**	Fixed	5.91	0.550	0
	BCC	5	**0.74**	**0.60–0.92**	**0.007**	Fixed	3.33	0.504	0
	SCC	3	0.90	0.70–1.15	0.387	Fixed	1.34	0.511	0
TT/TC versus. CC	Overall	15	0.94	0.81–1.09	0.424	Random	42.32	0	67.1
	melanoma	7	1.09	0.86–1.40	0.469	Random	23.25	0.001	74.2
	NMSC	8	0.84	0.70–1.00	0.053	Random	16.86	0.018	58.5
	BCC	5	0.85	0.65–1.12	0.241	Random	13.06	0.011	69.4
	SCC	3	**0.81**	**0.68–0.98**	**0.029**	Fixed	2.57	0.276	22.2

NMSC, nonmelanoma skin cancer; BCC, basal cell carcinoma; SCC, squamous cell carcinoma; N, number of studies; Fixed, fixed effect model; Random, random effect model; P_a,_ test for association; P_b,_ test for heterogeneity.

### Test of Heterogeneity analysis

**Figure 6:**
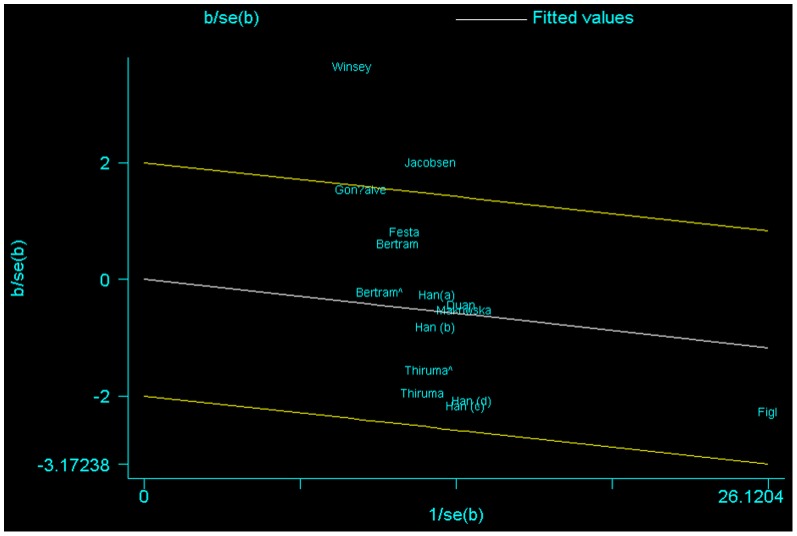
Galbraith plots of *XRCC3* C18067T polymorphism and skin cancer risk in dominant model TT/TC versus . CC. The studies of Winsey et al. and Jacobsen et al. were spotted as outliers.

There was significant heterogeneity for homozygote comparison (TT versus CC: P = 0.022, *I^2^* = 47.3%); heterozygote comparison (TC versus CC: P = 0.001, *I^2^* = 62.9%); and dominant genetic model (TT/TC versus CC: P = 0, *I^2^* = 67.1%). To explore the sources of heterogeneity, we performed metaregression and subgroup analyses. Metaregression analysis of data showed that the tumor type was the major source which contributed substantially to heterogeneity. The Ethnicity (Caucasian or Mixed), Source of control (Hospital-based or Population-based), Genotyping methods (PCR-RFLP or TaqMan or PCR-SSP), Quality score (≥12 or <12) or Sample size (≤500 subjects or >500 subjects) were not effect modifiers. Subsequently, we performed subgroup analyses stratified by tumor type. The heterogeneity really reduced because there was no significant heterogeneity after subgroup analysis by tumor type under homozygote comparison (TT versus CC). However, heterogeneity still existed in heterozygote comparison (TC versus CC) and dominant genetic model (TT/TC versus CC) (Table 3). To further investigate the heterogeneity, we performed Galbraith plots analysis to identify the outliers which might contribute to the heterogeneity. As a result, we found that the studies Winsey et al [34] and Jacobsen et al [32] were outliers in heterozygote comparison (TC versus CC), and dominant model (TT/TC versus CC) (Figure 6). All *I^2^* values decreased obviously and *P_Q_* values were greater than 0.10 after excluding the two studies Winsey et al and Jacobsen et al in all genetic comparison models in subgroup populations (date not shown). The significance of the summary ORs for *XRCC3* C18067T polymorphism in different comparison models in subgroup analyses were not influenced by omitting the two studies.

### Sensitivity Analysis

**Figure 7:**
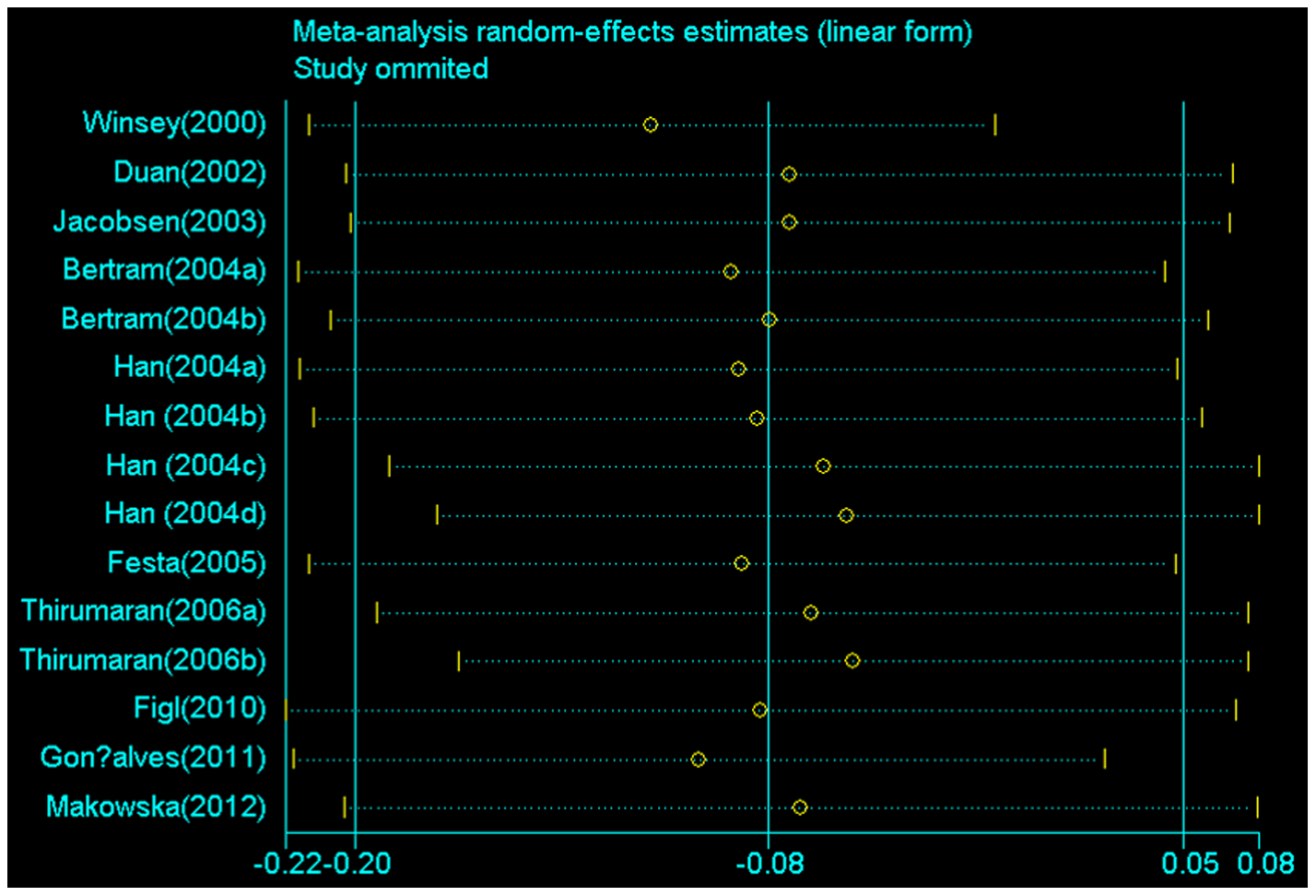
Sensitive analysis of *XRCC3* C18067T polymorphism and skin cancer risk (recessive model TT versus . TC/CC).

Sensitivity analysis was also used to evaluate the stability of the overall results by sequential omission of individual studies. In this meta-analysis, the result of sensitive analysis shows that any single study could not influence the overall results qualitatively, indicating robustness and reliability of our results (Figure 7).

### Publication Bias

**Figure 8:**
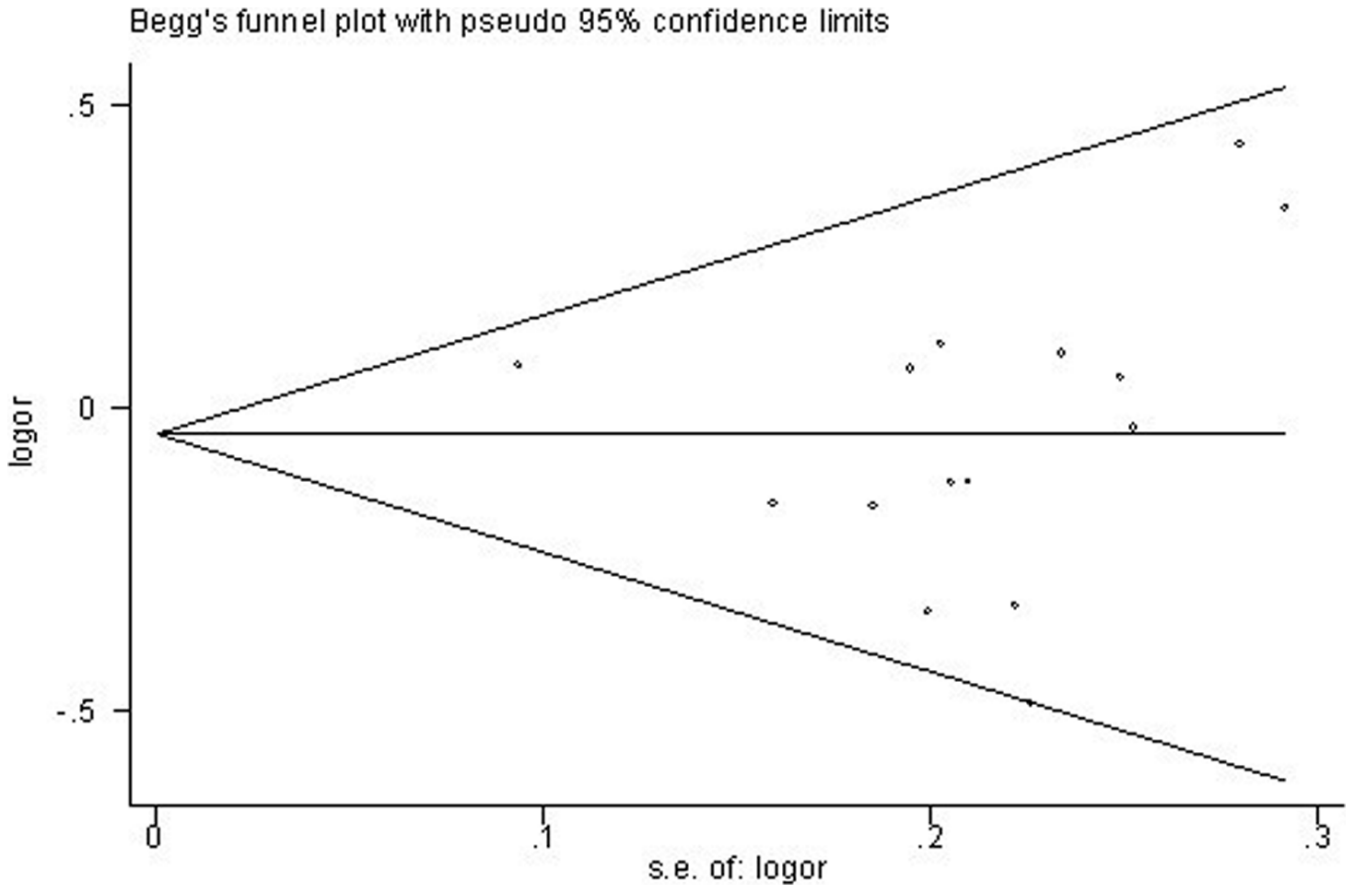
Begg's funnel plots for publication bias in the studies of the meta-analysis on the association between *XRCC3* C18067T and skin cancer risk of the overall populations (dominant model TT/TC versus .CC).

Begg funnel plot was created to assess the publication bias of literatures. Funnel plot of skin cancer risk associated with *XRCC3* C18067T polymorphism is shown in Figure 8. The shapes of the funnel plots did not reveal any evidence of obvious asymmetry. Then, the Egger test was used to provide statistical evidence of funnel plot symmetry. All the p values of Egger's tests were more than 0.05 and the results also suggested the absence of publication bias (t = 2.26; P = 0.596 for TT versus CC; t = 2.1; P = 0.056 for TC versus CC; t = 0.33; P = 0.748 for TT versus TC/CC; t = 0.33; P = 748 for TT/TC versus CC). All the above results suggested that publication bias was not evident in this meta-analysis.

## Discussion

DNA injury and repair plays a critical role in carcinogenesis. DNA repair pathways are of great importance in the removal of damages, repair of base alterations caused by UV radiation, recombination of homologous or nonhomologous end joining, and other injuries caused by many carcinogenic agents [35], [36]. The accumulated DNA damage may activate the carcinogenesis finally. Thus, an SNP which take place in the exon of DNA repair pathway genes may lead to the alteration of DNA repair capability, even susceptibility or risk to cancers. Therefore, numerous studies have been performed on SNPs on DNA repair genes and found significant association between these SNPs and cancer susceptibility [37]–[39].

The *XRCC3* in homologous recombination repair (HRR) pathway plays a very important role in DNA double-strand break repair (DSBR). Variations in the *XRCC3* gene might lead to altered protein structure or function which may change DSBR efficiency and result in cancer. The functional SNP of *XRCC3*, rs861539 (C>T) which causes a substitution of corresponding amino acid (threonine to methionine) at codon 241, has been extensively investigated. Matullo et al have demonstrated that the *XRCC3* C18067T polymorphism was associated with DNA repair capacity, which made it well-founded to hypothesize that *XRCC3* C18067T polymorphism may be associated with cancer risk [40]. Up to now, a lot of studies have been conducted to investigate the relationship between *XRCC3* C18067T polymorphism and skin cancer risk. Unfortunately, the results of these previous studies have been inconsistent. Unfortunately, so far there has not yet a report which comprehensively and specially evaluates all of the previous literature to get a precious estimate of this association between *XRCC3* C18067T polymorphism and skin cancer risk.

It is well recognized that small genetic association studies could inevitably add the risk that chance could be responsible for their conclusions because they have different study designs, diverse genotyping methods, insufficient statistical power, different population lifestyle and background. However, meta-analysis has the advantage of reducing random error and achieving precise estimates for potential genetic associations through pooling data. To the best of our knowledge, no meta-analysis specially evaluating on the association between the *XRCC3* C18067T polymorphism and skin cancer risk has been performed, and the present meta-analysis is the first study on such an association. Consequently, fifteen individual case-control studies more than 10000 individuals (4329 cases and 7291 controls) were included in our meta-analysis.

So far, plenty of studies have evaluated the *XRCC3* C18067T polymorphism with cancers risk, including colorectal, bladder, lung, breast, pancreatic cancer and so on. And a few meta-analyses have been conducted on *XRCC3* C18067T polymorphism and cancers risk, including colorectal cancer, lung cancer, bladder cancer, and breast cancer. Our study was carried out to investigate the association between *XRCC3* C18067T polymorphism and skin cancer risk. Different results derived from previous meta-analysis which focused on relationship between *XRCC3* Thr241Met polymorphism with cancers risk. This phenomenon indicates that the *XRCC3* C18067T polymorphism exerts different effect on various types of cancers. So that it is necessary to get a better understanding of *XRCC3* C18067T polymorphism on skin cancer risk, especially when inclusive and controversial findings still exists. The present meta-analysis was carried out by critically reviewing 15 individual case-control studies on *XRCC3* C18067T polymorphism and skin cancer risk. Our meta-analysis showed *XRCC3* C18067T polymorphism was not associated with risk of skin cancer. Subgroup analysis based on tumor type indicated that *XRCC3* C18067T polymorphism was not associated with risk of cutaneous melanomas but with decreased risk of nonmelanoma skin cancer. Both basal cell carcinoma and squamous cell carcinoma are two main tumor types of nonmelanoma skin cancer. Therefore, we divided them into two groups for further analysis. As a result, we found that *XRCC3* C18067T polymorphism was associated with decreased risk of both basal cell carcinoma and squamous cell carcinoma.

In our study, the study of Figl et al accounted for more than 25% weight and its sample size was relatively large (1184 cases/1274 controls). Its conclusion showed no significant association between *XRCC3* C18067T polymorphism and cutaneous melanomas, which was consistent with our pooled conclusion. Moreover, deleting any study in sensitive analysis would not materially alter the corresponding pooled ORs, which reflects the reliability and stability of our study.

HWE is an important law of genetic equilibrium. It seems that selection bias may occur if the genotype distribution of *XRCC3* C18067T polymorphism among control populations disobeyed the law of HWE. It is widely accepted that deviation from HWE could be caused by genetic reasons including non-random mating, or the alleles which reflects recent mutations have not reached equilibrium and other methodological reasons [41], [42]. Because of all of the above reasons, the results of genetic association studies might be spurious if genotypes distribution in the control population were not consistent with HWE. In the current meta-analysis, the distributions of genotype among control groups of all eligible studies conform to HWE, suggesting HWE would not influence the final result substantially and would make our results more reliable.

The heterogeneity plays an important role when performing meta-analysis and finding the source of heterogeneity is very important for the final result of meta-analysis. Because the inconsistent findings included in our meta-analysis among different studies were probably attributed to different genetic backgrounds, environmental exposures, genotyping methods and sample size. In the current study, obvious heterogeneity between-study was found in the overall population. Through conducting meta-regression, we found that the heterogeneity could not be explained by several possible source of heterogeneity such as Ethnicity, Source of control, Genotyping methods or Sample size. However, we found the tumor type was the major source of the high heterogeneity in our meta-analysis. Therefore, we perform subgroup according to tumor types. However, the tumor types did not explain all heterogeneity in this meta-analysis and other sources need further investigating. It is possible that other limitations of recruited studies may partially contribute to the observed heterogeneity. For this reason, we conducted analyses using the random effects model. Another important aspect which may have a negative effect on our meta-analysis is the publication bias. In our meta-analysis, Funnel plot and Egger's test were used to test the publication bias of the included studies. Both the shape of funnel plot and statistical results show no obvious publication bias, this suggests that the publication bias have little effect on the results in our study and the results of our meta-analysis are relatively stable and reliable.

Although comprehensive analysis was conducted to show the association between *XRCC3* C18067T polymorphism and risk of skin cancer, there are still some limitations should be pointed out. Firstly, the primary studies in the present meta-analysis mainly provided data towards Caucasians. Given that the race-specific association probably exists, other ethnicities including Asians, mixed and Africans should be investigated in future studies. Secondly, only four of eleven eligible studies used controls that were population-based [28]–[30], [32]. Other articles used hospital-based controls, which may not be representative of the general population. Thirdly, subgroup analyses according to age, gender, radiation exposure, and other confounding factors haven't been performed in the study because no sufficient relevant data available in the primary studies.

In spite of the shortages above, our meta-analysis had also several advantages as follows: First, a meta-analysis of the association of *XRCC3* C18067T polymorphism on skin cancer risk is statistically more powerful than any other single study. Second, strict searching strategy which combination computer-assisted with manual search make the eligible studies included as much as possible. Third, the quality of case-control studies included in the meta-analysis was met our inclusion criteria and was satisfactory, and the sensitivity analysis and publication bias analysis indicated the stability and credibility of the meta-analysis, which leads to a more convincing result. More important, the process of literature selection, data extraction and data analysis in the meta-analysis was well designed and conducted.

In summary, this meta-analysis systematically analyzed the association between *XRCC3* C18067T polymorphism and the risk of skin cancer. The pooled results suggest that the *XRCC3* C18067T polymorphism was not associated with risk of cutaneous melanoma but contributed a decreased risk to nonmelanoma skin cancer including basal cell carcinoma and squamous cell carcinoma. Considering the limited sample size and ethnicities included in the meta-analysis, further large scaled and well-designed studies are needed to confirm our results.

## Supporting Information

Checklist S1 **PRISMA checklist.**
(DOC)
